# Galectin Domain Containing Protein from *Haemonchus contortus* Modulates the Immune Functions of Goat PBMCs and Regulates CD4+ T-Helper Cells In Vitro

**DOI:** 10.3390/biom10010116

**Published:** 2020-01-09

**Authors:** Muhammad Ali-ul-Husnain Naqvi, Muhammad Ali Memon, Tahseen Jamil, Sana Zahra Naqvi, Kalibixiati Aimulajiang, Javaid Ali Gadahi, Lixin Xu, Xiaokai Song, Xiangrui Li, Ruofeng Yan

**Affiliations:** 1 MOE Joint International Research Laboratory of Animal Health and Food Safety, College of Veterinary Medicine, Nanjing Agricultural University, Nanjing 210095, China; 2017207047@njau.edu.cn (M.A.-u.-H.N.); 2016207040@njau.edu.cn (M.A.M.); 2018207077@njau.edu.cn (S.Z.N.); 2017207022@njau.edu.cn (K.A.); xulixin@njau.edu.cn (L.X.); songxiaokai@njau.edu.cn (X.S.); lixiangrui@njau.edu.cn (X.L.); 2 Sindh Agriculture University, Tandojam 70050, Sindh, Pakistan; tahseenjamil1992@gmail.com (T.J.); jagadahi@sau.edu.pk (J.A.G.)

**Keywords:** galectin, immunomodulation, PBMCs, apoptosis, T helper-9 cells, interleukin-9

## Abstract

Galectins are glycan-binding proteins that are widely expressed and distributed in mammalian tissues as well as cells of innate and adaptive immune responses. CD4+ T-helper cells differentiate into effector subsets in response to cytokines. T helper 9 cells are one of the recently described subsets of effector T cells that are relatively new and less studied. In this study, galectin domain containing protein from *Haemonchus contortus* (Hc-GDC) was cloned, expressed in pET32a, and immunoblotting was performed. Localization of recombinant (r)Hc-GDC on outer and inner surface of *H. contortus* worm and binding with goat Peripheral Blood Mononuclear cells (PBMCs) were performed using immunofluorescence assay. Moreover, effects of rHc-GDC on proliferation, apoptosis, cell migration, and the nitric oxide production in goat PBMCs were evaluated. Furthermore, modulatory effects of rHc-GDC on production of Th1, Th2, and Th9 cells were evaluated by flowcytometry and on interferon gamma, interleukin (IL)-4 and IL-9 were evaluated by quantitative real-time polymerase chain reaction. The results demonstrated that rHc-GDC was successfully cloned, expressed in expression vector as well as in the gut surface of adult *H. contortus* worm and successful binding with PBMCs surface were observed. Immunoblotting results revealed that rHc-GDC is an important active protein of *H. contortus* excretory and secretory products. Moreover, the interaction of rHc-GDC with host cells increased the production of Th2, Th9 cells, IL4, IL-9, PBMC proliferation, nitric oxide, and cell migration. No effects of rHc-GDC were observed on PMBC apoptosis, production of Th1 cells, and secretions of IFN-γ and IL-10 cytokines. These findings indicate that recombinant GDC protein from *H. contortus* modulates the immune functions of goat PBMCs and has the potential to enhance protective immunity by inducing T helper-9-derived IL-9 in vitro.

## 1. Introduction

Galectins, animal lectins, are known to be widely distributed from lower invertebrates, such as nematodes and sponges, to higher vertebrates [1]. It has been demonstrated that the interaction of galectin with its receptor on the target cell governs the function of the galectin [2]. Galectins are found in various species but only 15 mammalian sourced galectins have been identified until now. Galectins are widely involved in mammalian innate and adaptive immune responses [3,4,5,6,7]. All galectins contain conserved carbohydrate-recognition domains (CRDs) of about 130 amino acids that are responsible for carbohydrate binding and may influence cellular functions through protein to protein interactions with other nuclear and cytoplasmic proteins. Moreover, galectins also play important roles in diverse pathological and physiological processes, including immune and inflammatory responses, by maintaining T cell homeostasis [8]. The major function of the immune system is to detect and abolish pathogenic parasites, microorganisms, and cancer cells [9]. Galectins have a unique ability to bind with glycans present on the surface of pathogenic parasitic worms [10]. Previously, galectin-like proteins have been identified in *Onchocerca volvulus* [11], *Teladorsagia circumcincta* [12], and *Haemonchus contortus* [13].

Peripheral blood mononuclear cells (PBMCs) contain various immune cells including T cells, B cells, dendritic cells, and natural killer cells, which play important roles in the immune system. CD4+ T-helper cells differentiate into effector subsets including Th1, Th2, Th17, and Th-9 in response to cytokines and receptor interactions from cell to cell contact. Th-9 cells are one of the recently described subsets of effector T cells that are not well known [14]. In essence, the signature cytokine for Th9 cells is interlukin-9 (IL-9), a pleiotropic cytokine with diverse functions [15,16,17,18]. The function of Th9 subtype has been associated with a wide range of inflammatory diseases [19]. In humans, Th9 cells play a protective role against tumors, allergy, asthma, and autoimmune diseases [20]. Moreover, different roles of Th9 cells have been reported in animal models during helminth enteric infection [21]. Detecting antigen-specific Th9 cells greatly relies on the value of lead cytokine produced by these cells following antigen exposure. Parasitic nematodes have developed complex mechanisms to contribute in host immunomodulation [22]. In previous studies, expression of IL-9 in T cells isolated from *Leishmania major*, *Trichuris muris*, and *Schistosoma mansoni*-infected mice was reported [23,24,25].

*Haemonchus contortus* is one of the most important cosmopolitan trichostrongylid parasites of ruminants which causes huge economic losses by compromising the productivity [26]. *H. contortus* inhabits abomasum and a highly fecund parasite. A single parasite of this nematode may cause 0.5 mL blood loss per day leading to acute anemia, edema, and even death in severe affected young animals [27]. Currently, the control of *H. contortus* infection is anthelmintic based, but frequent use of these products produce drug resistance in strongylid parasites. Thus, the identification of new preventive strategies is urgently needed. A vaccine may be the best alternative to control this infection effectively. Different nematodal immunomodulatory molecules, Excretory and Secretory Products (ESPs), have been proposed as vaccine candidates that influence the immune and cytokine function [28]. ESPs are produced and released by the parasites during infections. ESPs react to the molecules on the surface of the host cell to form receptor–ligand complexes and activate immune response. In our previous study, immunomodulatory effects of ESPs from *H. contortus* on Th9 cells and associated immune response have been identified (data not published). Galectin domain containing protein (GDC) from *H. contortus* is one of the ESPs that can be isolated from different larval stages of this nematode [26] and may be a potential candidate to induce protective immunity. To our knowledge, no study has been reported so far on the immunomodulatory effects of this protein on host immune functions.

In this study, the *GDC* gene was cloned, expressed, and confirmed through Western blotting. Localization of rHc-GDC in adult *H. contortus* worm and binding of recombinant Hc-GDC with goat PBMCs were also performed. Moreover, modulatory effects of recombinant GDC (rHc-GDC) on goat PBMC proliferation, cell migration, and nitric oxide production were evaluated. This is a novel approach to also investigate the antigen specific effects on the production of Th9 cells and IL-9 cytokine. Intracellular cytokine staining in conjunction with flow cytometry is one frequently used approach to accomplish this goal [29].

## 2. Materials and Methods

### 2.1. Ethical Statement

All experimental protocols were approved by the Science and Technology Agency of Jiangsu Province (Approval ID: SYXK (SU) 2010-0005).

### 2.2. Animals and Parasites

Local crossbred 5–6 months old goats were purchased from the local market of Jiangsu province and housed in an animal house of Nanjing Agricultural University under controlled conditions. Levamisole (8 mg/kg body weight) was orally administered to all goats to remove natural parasitic infections at two-week intervals. Microscopic analysis was performed for detection of helminth eggs in feces samples collected at week intervals. Furthermore, infected larvae of *H. contortus* (L3) were obtained from experimental infection by a conventional method [26]. Briefly, feces from *H. contortus* infected-goat were collected, crushed, mixed with water, and combined with vermiculite to make the mixture moist. The pan was covered with aluminum foil having several holes to allow air flow and put at room temperature for ten days. The larvae mixture was filtered through cheesecloth to recover larvae, which were identified microscopically and preserved at 4 °C in penicillin G treated water until use.

Female Sprague Dawley rats (body weight: 150 g) (*n* = 6) were purchased from the Experimental Animal Center of Jiangsu, PR China (Certified: SCXK 2008-0004). To collect polyclonal antibodies, rats were divided into experimental (*n* = 3) and control (*n* = 3) groups and housed in sterilized experimental animal house Nanjing Agricultural University.

### 2.3. Cloning and Sequence Analysis of Hc-GDC

Total RNA was extracted from adult *H. contortus* parasites using Trizol reagent (Invitrogen, Shanghai, China) as described previously [30]. Briefly, the cDNA was synthesized using cDNA Kit (Takara Biotechnology, Dalian, China) as per the manufacturer’s instructions and stored at −20 °C until use. The complete ORF (open reading frame) of GDC was amplified by RT-PCR (reverse transcription-polymerase chain reaction) using specific primers designed from CDS (conserved domain sequences) of *H. contortus* (Genbank: CDJ98063.1). The sequence of specific primers carrying *EcoR* I and *Hind* III digestion site was as follows: Sense Primer: (5′-GAATTCATGTCTTGCGTTCAATTCAG -3′) and antisense primer: (5′-AAGCTTTTAGCCGATATGAATAGAATGC -3′). The total 25 μL PCR product was made up of 12.5 μL 2× Taq Master Mix (Takara Biotechnology), 2 μL of cDNA, 8.5 μL ddH_2_O, and 1 μL of each primer. PCR amplification was performed as follows: initial denaturing (1 cycle) 94 °C for 5 min, followed by 35 cycles of 94 °C for 30 s, 55 °C for 30 s and 72 °C for 1–2 min, and finally extension at 72 °C for 10 min. E.Z.N.A. Gel Extraction Kit (Omega Bio-tech, Norcross, GA, USA) was used according to instructions of manufacturer to purify the PCR products and followed by ligation into cloning vector, pMD19-T (Takara, Dalian, China). Transformation of recombinant plasmid (pMD19-T/GDC) into *E. coli* DH_5α_ strain (Invitrogen) was performed and cultured in ampicillin containing Luria Bertini (LB) medium. The recombinant plasmid, pMD19-T/GDC, was identified by restriction enzyme digestion with *EcoR* I and *Hind* III. GDC gene was sub-cloned into pET32a, followed by confirmation of successful insertion in the proper reading frame by sequencing (Invitrogen) and blast online.

### 2.4. Purification of Recombinant Hc-GDC

The recombinant plasmid (pET32a (+)/Hc-GDC) was transformed into *E. coli* BL21 and cultured in ampicillin (100 μg/mL) containing LB at 37 °C. Protein was expressed by adding 1 mM isopropyl-β-d-thiogalactopyranoside (IPTG; Sigma-Aldrich, Shanghai, China) until the OD_600_ of the culture reached 0.6 [30] The cells were collected by centrifugation and lysed using lysozyme (10 μg/mL) (Sigma-Aldrich) and sonicated. The cell sonicated product was analyzed by 12% (*w*/*v*) sodium dodecyl sulfatepolyacrylamide gel electrophoresis (SDS-PAGE). The rHc-GDC protein was purified by Ni2+-nitrilotriacetic acid (Ni-NTA) column according to the manufacturer’s instructions. After that, the His-tagged protein was washed by elution buffer (300 mM NaCl, 40 mM NaH2PO4, pH 8.0) containing 400 mM of imidazole. The purity of purified rHc-GDC (12 μL) was analyzed by 12% SDS-PAGE followed by Coomassie blue staining. The purified rHc-GDC was refolded by renaturation buffer (20 mmol/L Tris-Cl, 500 mmol/L NaCl, 1 mmol/L GSH, 0.1 mmol/L GSSG, pH 8.0) containing different concentrations of urea (8, 6, 4, 2, 0 M) [31]. The concentration of refolded rHc-GDC protein was determined by Bradford procedure [32]. The rHc-GDC protein was detoxified using ToxinEraserTM Endotoxin Removal Kit (GeneScript, Piscataway, NJ, USA).

### 2.5. Generation of Polyclonal Antibodies

Complete Freund’s adjuvant mixed equally with rHc-GDC (300 μg) was injected subcutaneously in SD rats of experimental group to obtain polyclonal antibodies. After 2 weeks, three more doses of this protein mixed with Freund’s incomplete adjuvant were injected at a 1-week interval. After one week of the last dose, SD rats were anesthetized with 25% isoflurane (inhaling anesthesia) by an open drop method [33] and blood samples were collected from the eye. Finally, SD rats were euthanized by head dislocation. The serum samples from the experimental group and control group were prepared and stored at −80 °C until use.

### 2.6. Western Blot Assay

Western blotting played a preliminary role in selection of target protein to evaluate the immunogenicity and immuno-reactivity of antigen [34]. Antigenic characteristics of rHc-GDC were also evaluated in sera of *H. contortus* infected goats as described previously [35,36]. Firstly, purified rHc-GDC was separated on 12% SDS-PAGE and semi dry system (Novablot Hoefer, Holliston, MA, USA) was used to transfer onto PVDF (polyvinyl difluoride membrane, Millipore, Billerica, MA, USA) with a transfer solution (Tris 48 mM, glycine 39 mM, SDS 0.0375%, methanol 20%). Blocking buffer (5% skimmed milk) diluted in TBS-T (Tris-Buffered Saline containing 0.05% Tween 20) was used to block the membrane at 37 °C for 2 h. The PVDF membrane was washed three times, cut into strips and incubated at 37 °C with 1:100 diluted primary antibody (anti-*H. cortortus* goat serum/anti-rHcGDC polyclonal antibody) for 2 h. After another three washes with TBS-T, the strips were incubated with 1:4000 diluted secondary antibodies (Horseradish Peroxidase conjugated rabbit anti-goat IgG; Sigma, Hilden, Germany). Finally, immunoreactions were observed with the help of chromogenic substrate, 3-diaminobenzidine tetrahydrocholoride (DAB; Tiangen Biotech, Beijing, China).

### 2.7. Localization of rHc-GDC in Adult *H. contortus* Worms

*H. contortus* adult worms were fixed on glass slides (poly-l-lysine hydrobromide) with 4% formaldehyde-0.2% glutaraldehyde in PBS for 45 min and suspended in TISSUE-TEK^®^ O.C.T. compound (Sakura, Torrance, CA, USA) and blast freeze in liquid nitrogen. Worms were cut into pieces (10-µm thickness) with a cryotome (CM1950, Wetzlar, Germany) and washed with PBS. The slides were treated with 5% Bovine Serum Albumin (BSA) to block non-specific bindings followed by incubation with 1:100 dilutions of rat-anti-rHc-GDC antiserum (experimental group) and normal rat serum (control group) as first antibody at 37 °C. After 2 h incubation, slides of both groups were incubated with 1:3000 dilutions of Cy3-labeled Goat Anti-Rat as second antibody (Beyotime, Shanghai, China) at 37 °C for 1 h. DAPI (diamidino-2-phenylindole) was used to stain the nucleus of worm cells and anti-Fade Fluoromount Medium (Beyotime, Shanghai, China) was used before observing under confocal laser scanning microscope to prevent fading.

### 2.8. Separation of PBMCs

PBMCs were separated from collected goat blood by standard Ficoll-hypaque (GE Healthcare, Munich, Germany) gradient centrifugation method as described previously [37]. After washing twice with Ca2+/Mg2+-free PBS (pH 7.4), PBMCs were adjusted to the required density (1 × 10^6^ cell/mL) in culture medium (Roswell Park Memorial Institute 1640) containing 10% heat-inactivated fetal bovine serum (FBS), 100 U/mL penicillin or 100 mg/mL streptomycin (GIBCO, Paisley, UK). The trypan blue exclusion test was performed to evaluate the cell viability as described previously [38].

### 2.9. PBMC Binding Assay

Immunofluorescence assay was performed to evaluate the binding ability of rHc-GDC to goat PBMCs as described previously [30]. Briefly, fresh goat PBMCs were inoculated with 10 μg/mL rHc-GDC and PBS separately in a 24-well plate (1 mL/well). Plate was incubated at 37 °C in a humidified atmosphere with 5% CO2 for 1 h. Washed cells were permitted to settle on poly-L-lysine coated glass slides for 20 min and fixed with 4% phosphate-buffered paraformaldehyde at room temperature for 30 min. The slides were blocked with PBS containing 5% BSA at 37 °C for 1 h. Subsequently, slides were incubated with 1:100 dilution primary antibodies, rat anti-rHc-GDC sera, and normal sera (control slide) for two hours. After three washes, slides were incubated in dark with secondary antibodies, and rat anti-goat IgG coupled with Cy3 (Beyotime Institute of Biotechnology, Shanghai, China; 1:1000 dilutions) for 30 min. DAPI (Sigma, St. Louis, MO, USA) was subsequently added and slides were incubated for 5 min. Finally, after washing, slides were covered with coverslip and immersed in Anti-Fade Fluoromount solution (Beyotime Institute of Biotechnology, Shanghai, China). PBMCs were observed by confocal microscope with laser scanner (PerkinElmer, Waltham, MA, USA) at 100× magnification and digital pictures were taken using Nikon microscope software packages (Nikon, Tokyo, Japan).

### 2.10. Cell Proliferation Assay

Cell proliferation assay was performed in triplicate using cell counting kit-8 (CCK-8) assay reagent (Beyotime Biotechnology, Jiangsu, China) as reported previously [2]. Briefly, fresh goat PBMCs (1 × 10^6^ cells/mL) were seeded into 96-well plates and incubated with consecutive concentrations of rHc-GDC (10, 20, 40 and 80 μg/mL), PBS (Negative control), and purified pET32a protein (Positive control; 10 μg/mL) at 37 °C in a humidified atmosphere with 5% CO2 for 72 h. Before measuring the absorbance value (OD_450_) in the micro plate reader (Thermo Scientific, Minneapolis, MN, USA), 10 μL of the CCK-8 reagent was added in each well for 4 h.

### 2.11. Cell Apoptosis Assay

Fresh goat PBMCs (1 × 10^6^ cells/mL) were cultured into 24-well plates and incubated with consecutive concentrations of rHc-GDC (10, 20, 40 and 80 μg/mL), PBS, and purified pET32a protein (10 μg/mL) at 37 °C in a humidified atmosphere with 5% CO2 for 24 h. After washing, cells were re-suspended in binding buffer, and Annexin V-FITC (Miltenyi Biotec, Bergisch Gladbach, Nordrhein- Westfalen, Germany) was added to the cells and incubated for 15 min in the dark at room temperature [39]. Flow cytometer (BD Biosciences, San Jose, CA, USA) was used after addition of Propidium Iodide (PI, Sigma-Aldrich, Shanghai, China) to the cell suspension.

### 2.12. Cell Migration Assay

The migration assay was performed in triplicate using Trans-well system (Merck-Millipore, Boston, MA, USA), that allow PBMCs to migrate through polycarbonate membrane (8 μm pour size) as described earlier [40]. Goat PBMCs (1 × 10^6^ cells/mL) were scattered into a 24-well plate (1 mL/well) and incubated with different concentrations of rHc-GDC (10, 20, 40 and 80 μg/mL), PBS (Negative control), and purified pET32a protein (Positive control; 10 μg/mL) at 37 °C in a humidified atmosphere with 5% CO2 for 24 h.

### 2.13. Nitric Oxide Production Assay

Goat PBMCs (1 × 10^6^ cells/mL) were washed twice and 100 μL of cells were incubated with serial concentrations of rHc-GDC (10, 20, 40 and 80 μg/mL), PBS and purified pET32a protein (positive control), and in Dulbecco’s Modified Eagle Medium in 96-well plate at 37 °C in a humidified atmosphere with 5% CO2. Nitric oxide (NO) production assay was performed in triplicate by using Griess assay as the instructions given by Total Nitric Oxide Assay Kit (Beyotime Biotechnology, Jiangsu, China). The absorbance values of colored solution were measured using a micro plate reader at 450 nm (OD_540_) and converted these values to micro moles per liter (μmol/L) with a standard curve obtained by adding 0–80 (μmol/L) of sodium nitrate in fresh culture media. This experiment was performed in triplicate individually.

### 2.14. Determination of Cytokine Expression

Quantitative real-time polymerase chain reaction (QRT-PCR) was used to determine the expression of IFN-γ, IL-4, and IL-9. Initially, collected goat PBMCs were activated with 10 ng/ml phorbol myristate acetate (PMA) and 1 μg/mL ionomycin (Sigma) and treated with different concentrations of rHc-GDC (10, 20, 40, 80 μg/mL), PBS (Negative control) and purified pET32a protein (10 μg/mL). Subsequently, total RNA was extracted using Trizol reagent (Invitrogen, Shanghai, China) as described above and cDNA was synthesized using cDNA Kit (Takara Biotechnology, Dalian, China). Actin-β was used as reference gene ad sequence details of primers used for QRT-PCR are given below (Table A1 in Appendix A).

### 2.15. Intracellular Cytokine Staining and Flow Cytometry

The expression of markers on T helper cells from goat PBMCs, activated with 10 ng/mL Phorbol Myristate Acetate (PMA; Sigma-Aldrich, St. Louis, MO, USA) and 1 Mm ionomycin (Sigma-Aldrich), were determined by flow cytometry [41] after treated with different concentrations of rHc-GDC (10, 20, 40, 80 μg/mL), PBS (negative control) and purified pET32a protein (10 μg/mL) in 24-well plate containing RPMI 1640 medium at 5% CO_2_ 37 °C for 48 h. About 4–6 h before intracellular staining, protein transport inhibitor, Brefeldin A Solution (BFA; 10 μg/mL; BD Biosciences) was added [32]. Subsequently, cells were transferred to 1.5 mL tubes, centrifuged at 1500 rpm for 10 min, washed three with PBS and stained with anti-goat-specific antibodies conjugated with FITC, PE, and Percp-cy5.5 for 30 min at 4 °C. These goat Abs included anti-CD2, CD4, IL-4, IFN-γ, IL-9, and IL-10 were purchased from BD Biosciences (Franklin Lakes, NJ, USA). After that, cells were centrifuged at 500 rcf for 5 min at 4 °C, added 500 μL of fixation buffer (Beijing Solarbio Science and Technology Co., Beijing, China) and put at dark place for 20 min. After another washing, cells were permeabilized twice with BD Perm/Wash buffer (Becton Dickinson Biosciences, Franklin Lakes, NJ, USA). Cells were stained with intracellular cytokine antibodies, IL-9 and IL-10 (Becton Dickinson Pharmingen) in addition to evaluating the expression of Th9 cells. Finally, flowcytometry was performed acquiring the gate at 100,000 on FACS Canto II flow cytometer (Becton Dickinson) [42].

### 2.16. Statistical Analysis

Statistical analysis of the data was evaluated by using the Graph Pad Prism™ version 6.01software. All data obtained from the above experiments were displayed as mean ±SEM. One-way ANOVA followed by Tukey’s *post-hoc* test was employed to compare the variances between groups and considered statistically significant at * *p* < 0.05, ** *p* < 0.01, *** *p* < 0.001. QRT-PCR data were analyzed based on raw cycle threshold (Ct), obtained from the ABI Prism 7500 software (Applied Biosystems, Foster City, CA, USA) by the comparative Ct (2^−ΔΔ Ct^) method [43]. Flowcytometry data was analyzed using Cytometrists Expert software.

## 3. Results

### 3.1. Molecular Cloning, Expression, and Purification of Hc-GDC

**Figure 1:**
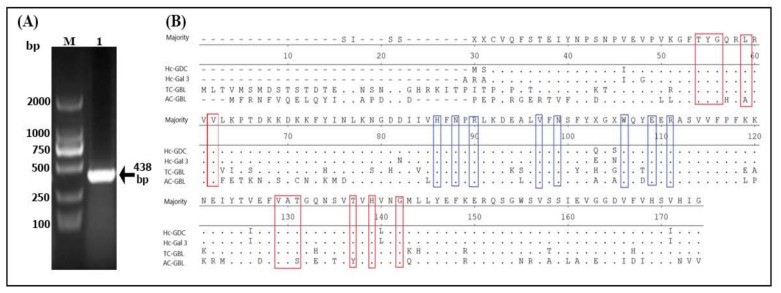
Cloning and multiple sequence alignment of amino acids rHc-Galectin Domain Containing. (**A**) the PCR generated fragment of GDC gene with molecular size of 438 bp, was obtained from *H. contortus* cDNA. Lane M: DNA molecular marker; Lane 1: Amplified PCR product. (**B**) The identity of rHc-GDC protein sequence aligned with Galectin 3 of *H. contortus* (GenBank: AAD45606.1; Hc-Gal 3), Galactoside-binding lectin of *Teladorsagia circumcincta* (GenBank: PIO73875.1; TC-GBL) and Galactoside-binding lectin of *Ancylostoma caninum* (GenBank: RCN38869.1; AC-GBL) was analyzed by ClustalW Multiple sequence software. Putative alternate dimerization interfaces based on amino acids sequences were marked as red boxes and sugar binding pockets based on amino acids sequences were marked as blue boxes. The residue that matches the majority is indicated by “-”.

**Figure 2:**
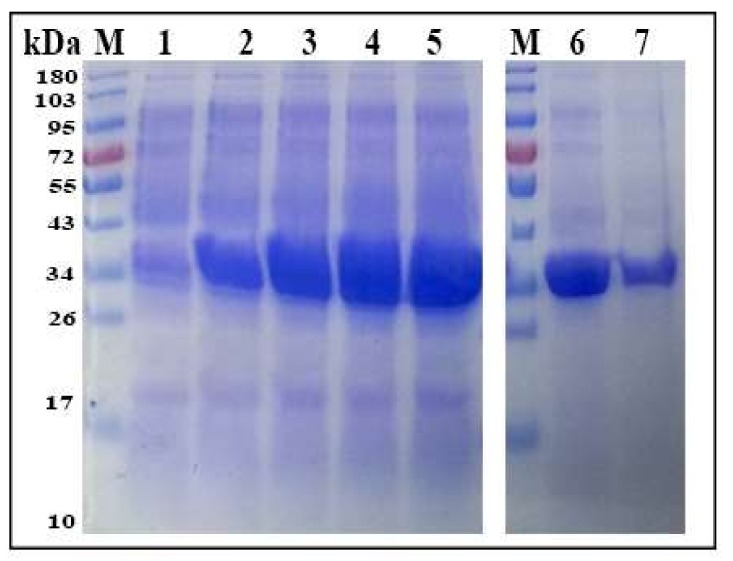
Expression and purification of rHc-GDC. Lane M: Standard molecular weight protein marker; Lane 1: Recombinant expression vector before induction of Isopropyl-β-d- thiogalactopyranoside (IPTG); Lane 2–5: Expression after IPTG induction at different time points; Lane 6: Pre-purified rHc-GDC; Lane 7: Purified rHc-GDC.

The fragment size of Hc-GDC gene (438) was detected between 500 and 250 (Figure 1A) and confirmed by sequence analysis. Multiple sequence alignment showed that all sequences were closely related with conserved domain residues (Figure 1B). Recombinant plasmid (pET32a + Hc-GDC) was induced with IPTG and analyzed by SDS-PAGE that showed the highest concentration after five hours of induction (Figure 2, Lane 5). The expressed protein showed a molecular weight of about 35 kDa (Hc-GDC, 14.6 ligated with pET32a, 20) after purification (Figure 2, Lane 7).

### 3.2. Western Blot Assay of rHc-GDC

**Figure 3:**
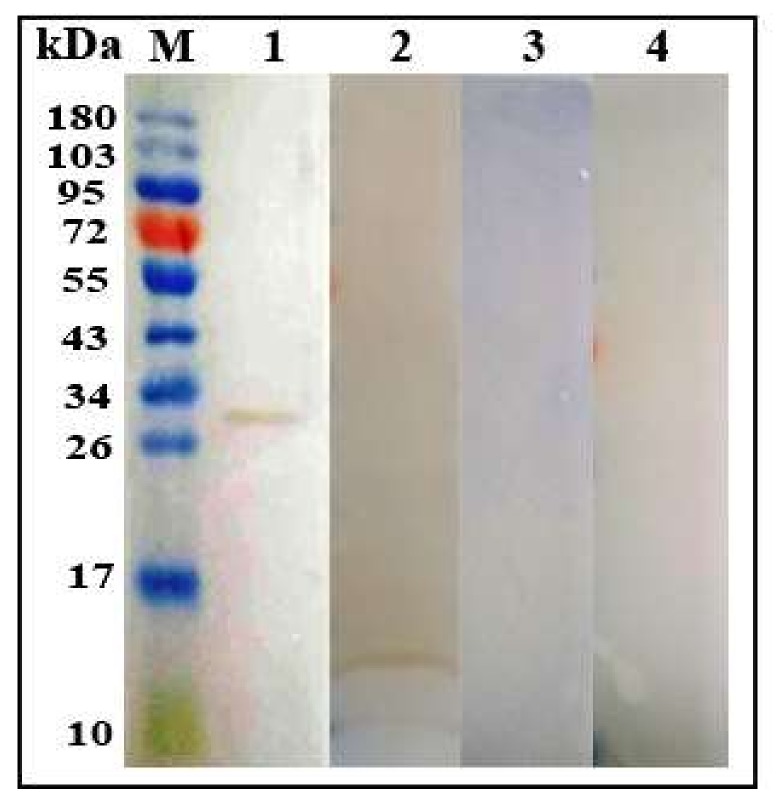
Western blot analysis of rHc-GDC. Purified rHc-GDC was electrophoresed by Gel Electrophoresis, stained with Coomassie blue, and transferred to a membrane for Western blot analysis. Lane M: Standard protein pre stain molecular weight Marker; Lane 1: Recombinant GDC protein detected by serum incubated with positive *H. contortus* goat; Lane 2: GDC extracted from *H. contortus* was detected by anti-rHc-GDC rat sera; Lane 3: No antibody detection was observed with sera of normal goat; Lane 4: No antibody detection was observed with sera of normal rat.

The purified His tagged fusion rHc-GDC protein was resolved on 12% SDS-PAGE and showed that rHc-GDC protein could be recognized by serum from goat experimentally infected with *H. contortus* kDa (Figure 3; Lane 1). Western blotting analysis revealed that rHc-GDC could be recognized by antibodies generated against rHc-GDC protein in SD rats’ sera. The molecular mass of the native GDC protein was 14.6 kDa, which was the same molecular weight as that of the rHc-GDC after reduction from pET32a protein (Figure 3; Lane 2). No protein was detected when membranes were incubated with normal goat sera (Figure 3; Lane 3) and normal rat sera (Figure 3; Lane 4). The results indicated that rHc-GDC had good antigenicity and it is one of the ESPs from *H. contortus* that could be recognized by the immune system of the host.

### 3.3. Binding of rHc-GDC to Goat PBMCs

**Figure 4:**
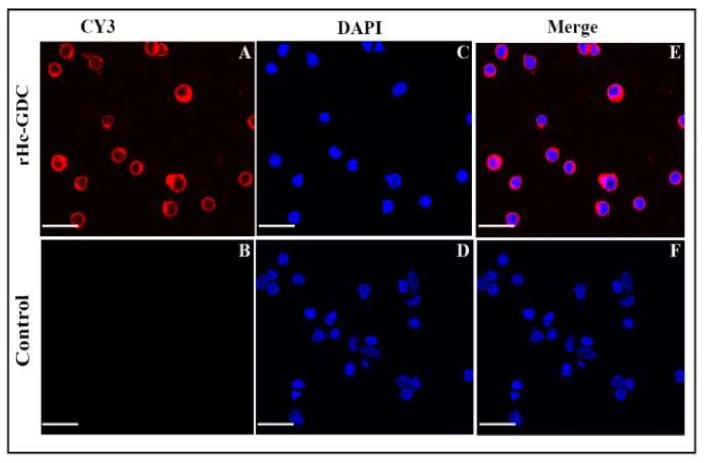
rHc-GDC protein binding to goat Peripheral Blood Mononuclear Cells (PBMCs) by immunofluorescence assay. Localization was performed by incubating PBMCs with rat anti-rHC-GDC IgG and negative rat IgG (control). (**A**,**B**) the target proteins (red) were visualized by Cy3-conjugated secondary antibody; (**C**,**D**) the nuclei of the corresponding cells were visualized by DAPI (blue) staining; (**E**,**F**) merge combination of red and blue channels. No protein binding was observed in the control group. Scale bar 40 μm.

The cultured goat PBMCs were analyzed by immunofluorescence assay (IFA) using a confocal microscope. DAPI was used to stain nuclei (blue fluorescence) and a secondary antibody was labeled with Cy3 to stain the cells (red fluorescence). Confocal microscopy results revealed that rHc-GDC was merged with the cell surface and showed red and blue combined fluorescence (Figure 4, upper Section), whereas no combined fluorescence was observed in cells treated with PBS (Figure 4, lower Section). The dense concentration of red around goat PBMCs revealed that rHc-GDC could strongly bind to the surface of the cell.

### 3.4. Localization of rHc-GDC on surface of Adult H. contortus Worm

**Figure 5:**
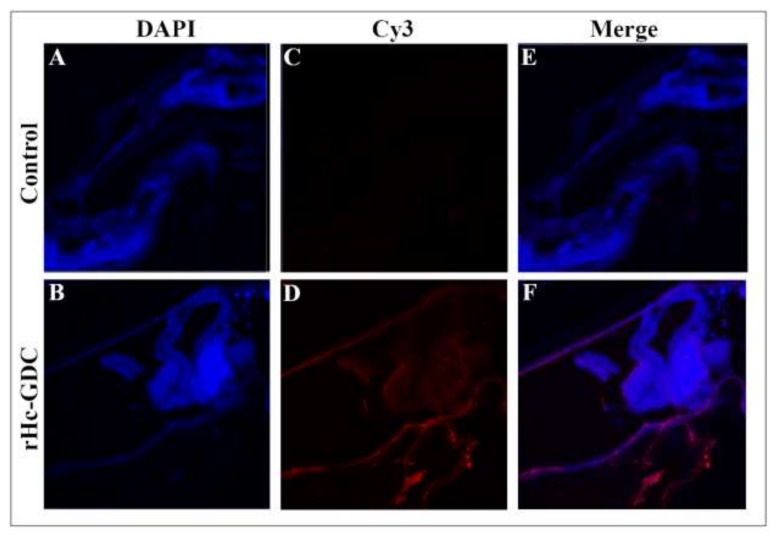
Expression of rHc-GDC protein in adult *H. contortus* by immunofluorescence assay. (**A**,**B**) nuclei stained with DAPI (blue); (**C**,**D**) target protein localization in adult worms using Cy3-conjugated secondary antibody; (**E**,**F**) merge image of DAPI and Cy3. No florescence was observed in control. Scale bar 40 μm.

Immunofluorescence assay was performed to detect localization of rHc-GDC using partial body sections of *H. contortus* adult. Clusters of blue spots inside the body of worms are indicating the position of nuclei along the gut structure in both merge and DAPI sections. IFA results indicated that Hc-GDC (red color) might be localized outer and inner surfaces of the membrane and in the gut region of the parasite as well. No protein was detected in the control group (Figure 5).

### 3.5. Effect of rHc-GDC on PBMC Proliferation

**Figure 6:**
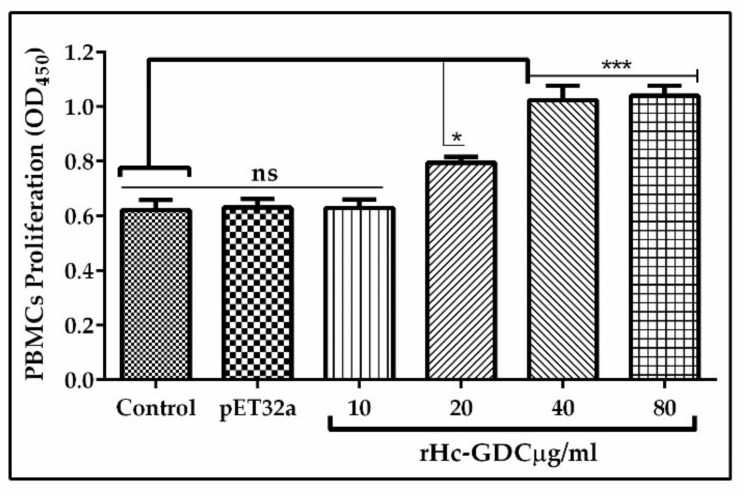
Effects of rHc-GDC on PBMC proliferation. PBMCs were treated with PBS (negative control), purified pET32a protein (positive control), and serial concentrations of rHc-GDC for 72 h. The cell proliferation index was calculated considering the OD_450_ values in PBS control as 100%. The data were analyzed from the standard error mean of three independent experiments (* *p* < 0.05, *** *p* < 0.001 and ns: no significant difference).

The effect of rHc-GDC on the multiplication of PBMCs was evaluated by incorporation of cell counting kit 8 (CCK8). The results of this assay indicated that there were no significant effects (ANOVA, F _(5, 12)_ = 29.83, *p* > 0.9999) between the 10 μg/mL rHc-GDC treated group, pET32a, and the negative control group. Significantly increased proliferation of PBMCs was observed in groups incubated with 20 μg/mL (ANOVA, F _(5, 12)_ = 29.83, *p* = 0.048), 40 μg/mL and 80 μg/mL (ANOVA, F _(5, 12)_ = 29.83, *p* < 0.001) concentration of rHc-GDC protein as compared to PBS (control) and pET32a (Figure 6).

### 3.6. Effect of rHc-GDC on PBMC Viability and Apoptosis

Cell viability assessed by means of the Trypan blue exclusion test was consistently >95%. To explore the impact of different concentrations of rHc-GDC on early and late apoptosis of PMBCs, cell apoptosis assay was performed. The results showed that there was no significant change between annexin V positive-purified pET32a protein and PBS (negative control). Moreover, there was no significant impact (*p* > 0.05) of rHc-GDC observed on the early and late apoptosis of goat PBMCs at concentrations of 10 μg/mL, 20 μg/mL, 40 μg/mL, and 80 μg/mL.

### 3.7. PBMC Migration Assay

**Figure 7:**
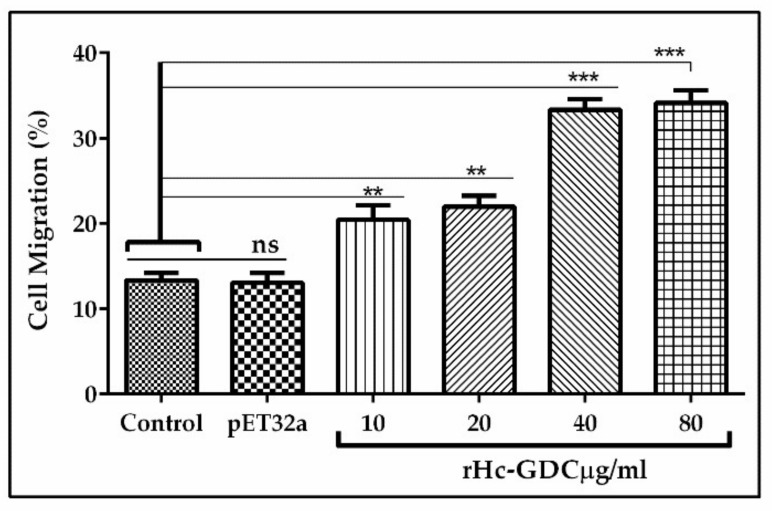
Effects of rHc-GDC on PBMC migration. Cells were treated with PBS (control), purified pET32a protein, and serial concentrations of rHc-GDC. Then, the random migration was determined. Statistical differences between standard error mean of three independent experiments (*n* = 5) were calculated using one-way ANOVA and marked ** when *p* < 0.01, *** when *p* < 0.001 and ns when no significant difference was found.

Cell migration assay was performed to evaluate the impacts of rHc-GDC on cell migration. The percentage of migrated cells through Millipore polycarbonate membrane (Millicell^®^ Cell Culture Inserts, Corning, NY, USA) into the lower chamber was calculated, and results indicated that 13.15 ± 1.02% with control and 13.08 ± 1.21% cells with pET32a group were migrated. Significant migration into a lower chamber was observed in a group treated with 10 μg/mL (20.61 ± 2.83%), 20 μg/mL (21.84 ± 3.21%), 40 μg/mL (32.44 ± 2.77%), and 80 μg/mL (34.5 ± 2.51%) protein concentration was observed as compared to the control and pET32a group (Figure 7).

### 3.8. Nitric Oxide Production Assay

**Figure 8:**
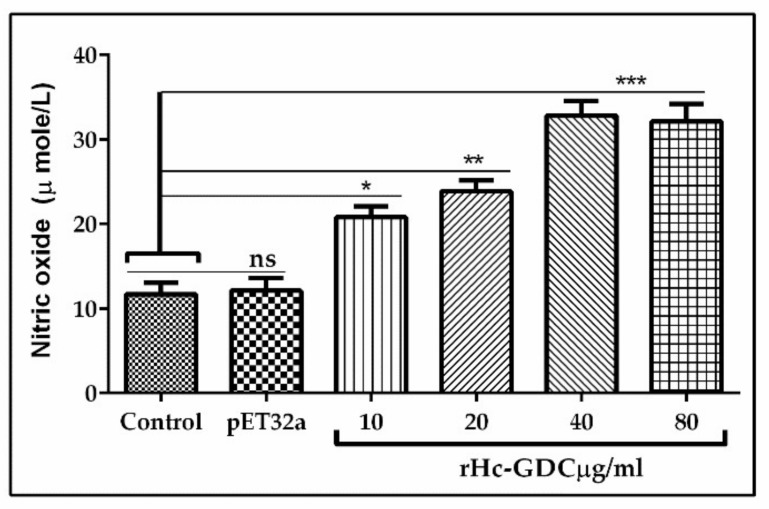
Influence of rHc-GDC on nitric oxide production by PBMCs in vitro. Cells were incubated with PBS, pET32a, and serial concentrations of rHc-GDC protein for 24 h in 37 °C and 5% CO_2_. The nitrate concentration in the PBMCs was measured by using the Griess assay. Statistical differences between standard error mean of three independent experiments were calculated using one way ANOVA and marked * when *p* < 0.05, ** when *p* < 0.01, *** when *p* < 0.001, and ns when no significant difference was found.

The total nitric oxide assay kit was used to evaluate the effect of rHc-GDC on nitric oxide production by PBMCs. Results revealed that the rHc-GDC with 10 μg/mL, 20 μg/mL, 40 μg/mL, and 80 μg/mL concentration showed significant (ANOVA, F _(5, 12)_ = 25.03, *p* < 0.001) NO production in PBMCs as compared to control group, whereas no significant difference in NO production was observed between control and pET32a (Figure 8).

### 3.9. Protein rHc-GDC Modulated Cytokine Secretion by PBMCs

**Figure 9:**
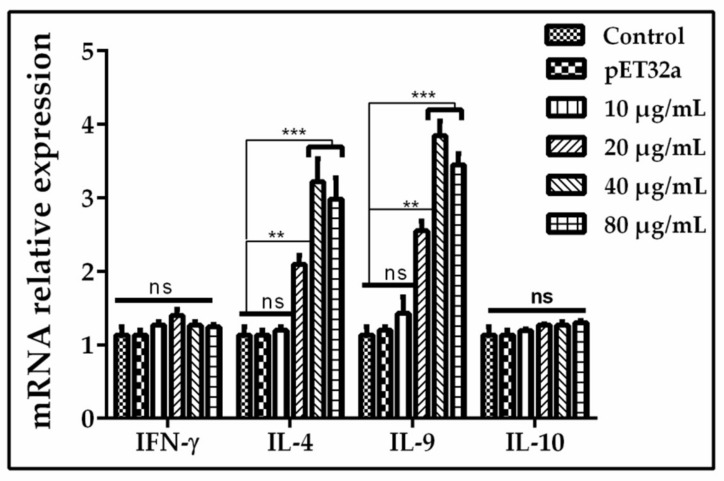
Relative expression of Interferon gamma, Interleukin (IL)-4, IL-9 and IL-10 cytokines in goat PBMCs stimulated by the recombinant Hc-GDC were evaluated. Cells were incubated with the recombinant GDC for 48 h, the mRNAs was quantified by QRT-PCR. The significant level was set at ** *p* < 0.01, *** *p* < 0.001, and ns non-significant compared with the untreated group (control). Data are representative of three independent experiments.

QRT-PCR assay was performed to assess the cytokine production by PBMCs that had been incubated with different concentrations of rHc-GDC. As shown in Figure 9, the production of IL-4 and IL-9 increased significantly (*p* < 0.001) when BFA treated PBMCs were cultured with 20, 40, and 80 μg/mL rHc-GDC, while the expression of IFN-γ and IL-10 did not show obvious changes (*p* > 0.05) when compared with purified pET-32a protein and PBS groups.

### 3.10. Effect of rHc-GDC on Th1, Th2, and Th9 Cell Differentiation

**Figure 10:**
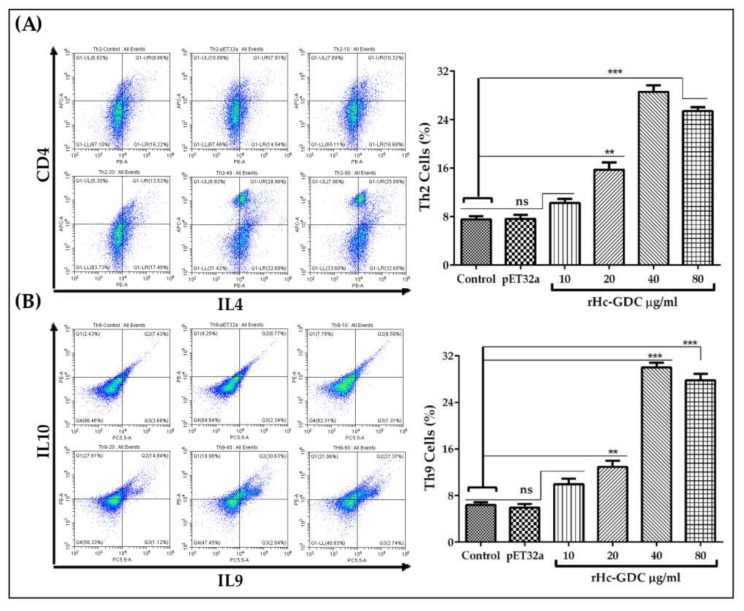
Effects of rHc-GDC on population of Th2 and Th9 cells by flow cytometry. (**A**) pseudo plot analysis (gated on CD4+ T cells) and proportion of PBMCs derived-Th2 cells treated with PBS (negative control), purified pET32a protein (positive control) and different concentrations of rHc-GDC (10, 20, 40, 80; μg/mL) using representative intracellular cytokine antibodies (CD4 and IL4); (**B**) pseudo plot analysis (gated on CD2+ CD4+ T cells) and proportion of PBMC derived-Th9 cells treated with PBS (negative control), purified pEt32a protein (positive control), and different concentrations of rHc-GDC (10, 20, 40, 80; μg/mL) using representative intracellular cytokine antibodies (IL-9 and IL-10). Data are presented as the standard error mean and representative of triplicate experiments (** *p* < 0.01, *** *p* < 0.001).

To evaluate the effect of rHc-GDC on Th1, Th2, and Th9 cells, different concentrations of rHc-GDC (10, 20, 40, 80; μg/mL) were incubated with goat PBMCs, and flowcytometry was performed by setting the gate on CD4+ IFN-γ, CD4+ IL4, and CD2+ CD4+ T cell (intracellular antibodies: IL-9 and IL10) were used for the Th1, Th2, and Th9 cell count, respectively. The rHc-GDC didn’t show any effect on the expression of Th1 cells (Data not shown). The rHc-GDC showed a potent capability to induce expression level of IL-4 and Il-9 cytokine; we further examined the impact of rHc-GDC on differentiation of Th9 cells, and found that the proportion of Th2 (Figure 10A) and Th9 cells (Figure 10B) were induced significantly (*p* < 0.001) by rHc-GDC in a dose-dependent manner. The flowcytometry results revealed that 40 μg/mL of rHc-GDC showed the highest percentage of Th9 (31.35%) production, while percentage production of 20 and 80 μg/mL was recorded as 13.02% and 27.86%, respectively. The data showed that 20, 40, and 80 μg/mL markedly increased as compared to the control group (0 μg/mL) while the group treated with 10 μg/mL rHc-GDC (10.05%) and pET32a group (6.89%) showed no significant difference when compared with the control (4.71%) group (Figure 10).

## 4. Discussion

Galectins are an evolutionarily ancient family of proteins which are closely related to carbohydrate binding proteins, located either intracellularly or extracellularly and can be produced by both the parasite and the host [44]. Previous studies suggested that parasite galectin might play an important role in host–parasite interactions [7]. Previously, the ability of recombinant galectins to exert various in vitro activities by engaging glycans on the extracellular matrices as well as cell surface has been convincingly and extensively documented [45,46]. In this study, recombinant GDC protein from *H. contortus* was characterized and localization of this protein by immunofluorescence assay confirmed the binding with host immune cells, in surface ligand complex shape, which is a characteristic feature of ESPs to modulate the immune functions of host PBMCs [47]. Mammalian galectins have been identified in various cells and tissue; however, the digestive tract is particularly rich in galectins [48]. Similar to its mammalian homologues, immunohistochemical investigation of this study also demonstrated that the native GDC protein was predominantly covering the internal gut surface of adult *H. contortus* worm. In our previous proteomic analysis, galectin was found at luminal surface of the adult worm of *H. contortus* [49]. In another study, a 32 kDa galectin was found most abundantly in the adult cuticle of *Caenorhabditis elegans* [50].

Proteomic analysis of ESPs indicated that galectins were expressed numerously in *H. contortus*, *Teladorsagia circumcincta*, and *Ostertagia ostertagi* [51]. In this study, we found that rHc-GDC could be recognized by the antiserum from goats experimentally infected with *H. contortus*. All of these findings indicated that the galectin domain containing protein of *H. contortus* is one of the excretory secretory antigens and interacts with the host immune system during infection. Galectins from the parasite source have evidenced an increased expression during the infection and hence they are considered as key players in host–parasite interactions [51,52].

In previous studies, immuno-regulatory roles of galectins have been reported in different cellular processes such as cell adhesion, cell proliferation [53], apoptosis [54], and immune responses [55]. Cell proliferation and migration are very important for the development of immune responses and tissue regeneration. Cell proliferation is regulated by both T cells and antigen-presenting cells (APCs) [56], and increases the number of immune effector cells. These effector cells migrate to the surrounding tissue or infection sites through the cell migration process and sometimes resulted in tissue damage [52,57]. In this study, it was identified that goat PBMC proliferation was increased at a significant level, which ultimately showed constant apoptosis of PBMCs in response to rHc-GDC treatment. Moreover, increased migration rate of immune effector cells was observed in vitro. The proliferation results of this study are in line with the results of the previous study [58]. In contrast, cell proliferation was suppressed after PBMCs were treated with rHcARF1 [59]. Specific immuno-regulatory properties of antigen might be the possible reason for these differences. The possible reason may be the dependency of migration on cell proliferation as cells need more room to migrate. Moreover, further study is needed to investigate the actual mechanism of cell migration.

Nitric oxide (NO) has been documented as an important immunomodulator in various infections including *H. contortus* by mediating host protection, parasite killing, or suppressing the growth. Previously, it was documented that NO had been involved in nonspecific defense mechanism against *H. contortus* and various other parasites [60]. In the present study, immunomodulatory effects of the rHc-GDC on the NO production by goat PBMCs were evaluated. Cells incubated with different concentrations of rHc-GDC significantly increased the NO production in a dose dependent manner. A constant increase in NO production indicated that rHc-GDC involved in the immunomodulatory regulation of NO on goat PBMCs. A previous study supports these results in which one of the ESPs, recombinant Arginine kinase, was used. Insights of NO production mechanism are still lacking [39].

PBMCs consist of several immune cells, including lymphocytes and monocytes that play important roles in innate and adaptive immunity [26]. The current study focused on evaluating the effects of rHc-GDC protein on PBMCs-derived CD4+ T helper cells. CD4+ T helper cells play an important role in the host defense of pathogens [19]. T helper cells mainly consist of Th1, Th2, Th17, and newly described Th9 cells. Th1 cells induce delayed-type hypersensitivity reactions and Th2 cells are excellent helpers for B-cell antibody secretion [14]. A previous study reported that T cell subsets are closely related to dyslipidemia and kidney injury during preeclampsia disease [61]. Moreover, reduced T cell activity has been reported during Parkinson’s disease [62]. Immune cells secrete cytokines that enable communication and regulation of the immune system [49]. Previously, increased levels of CD4+ T cells-derived IL-2 and IL-21 have been reported in inflammatory bowel disease [63]. Moreover, previous studies documented that IL-9 is a signature cytokine for Th9 cells with diverse functions [64,65]. In this study, PBMCs incubated with different concentrations (20, 40, 80; μg/mL) of rHc-GDC increased Th9 levels in a dose-dependent manner and, in turn, the surge of signature cytokine was recorded in vitro. The rHc-GDC didn’t show a significant effect on the production of suppressive cytokine IL-10, Th1 cells, and associated immune response IFN-γ, but the secretion of IL-4 cytokine and production of Th2 cells were up-regulated. One of the possible reasons is that Th2 cells in combination with IL-4 promoted the differentiation of Th9 cells directly. Th2 lymphocytes and mast cells also play important roles during nasal congestion, over sneezing, rhinorrhea, and itching [66]. The physiological function of IL-9 seems to be in some way connected with a Th2 response, as IL-9 is important in the immune defense against helminth infection but also appears during allergic reactions in the lung, both scenarios that depend on Th2 responses [67]. More recent studies revealed the multifunctional activities of IL-9 cytokine. IL-9 production can modulate immune response in both manners as IL-9 promotes allergic and inflammatory reactions by recruiting mast cells, macrophages, and eosinophils [68,69]. Conversely, IL-9 enhances the immunosuppressive activity of natural regulatory T-cells (16). Previously, promotion of mast cells expansion was reported by IL-9 during some allergic responses in the lungs [70,71,72]. Similarly, in food allergy, effects of IL-9 production were also reported in mastocytosis and intestinal permeability [73]. Additionally, involvement of IL-9 has also been observed in the development of autoimmune diseases and its deficiency lessens the disease progression [68,74]. On the contrary, IL-9Rα-deficient mice had developed more severe experimental autoimmune encephalomyelitis (EAE) [75]. In addition, high levels of IL-9+TH17 cells were found in diabetic patients’ blood [76]. Furthermore, parasitic removal from the gastro intestinal tract can be promoted by IL-9. In these conditions, it is thought that Th9 cells produce IL-9 that stimulate the mucosal mast cells production that contribute in mucus production and increase the muscle contraction as well as intestinal permeability [21,77], while IL-9-deficient mice during lung parasitic infection caused by *Schistosoma mansoni* only decreased the mucus production and no effect on granuloma formation for parasitic egg removal [78]. As far as the tissue grafting is concerned, IL-9 increases the capability of T Regulatory cells for reduction of immune responses at the graft site [64,79]. The functions of IL-9 cytokine in immunity are still controversial and further studies are needed to understand it completely.

## 5. Conclusions

This study revealed that rHc-GDC is an important active protein of *H. contortus* ESPs that played crucial roles in the immune regulations. Results indicated that the interaction of rHc-GDC with host cells increased the production of Th9 cells, signature cytokine IL-9, nitric oxide, and cell migration in dose-dependent manner. The findings concluded that rHc-GDC has the potential to enhance the protective immunity by inducing Th2 and Th9 immune response in vitro, which will help to elucidate the host–parasite interaction. However, further studies are needed to evaluate the effects of rHc-GDC on Th9 immune response during different stages of *H. contortus* infection in goats.

## Appendix A

**Table A1:** Primer sequences for Quantitative real-time PCR.

Gene Name	Forward 5 → 3	Reverse 5 → 3	Amplification Size (bp)	Amplification Efficiency (%)
Beta-Actin	CACCACACCTTCTACAAC	TCTGGGTCATCTTCTCAC	106	95.41
IFN-γ	GAACGGCAGCTCTGAGAAAC	GGTTAGATTTTGGCGACAGG	131	98.02
IL-4	GTACCAGCCACTTCGTCCAT	GCTGCTGAGATTCCTGTCAA	148	97.11
IL-9	GATGCGGCTGATTGTTT	CTCGTGCTCACTGTGGAGT	103	98.68

Amplification efficiency (%) = (10^−1/slope^ − 1) × 100.
